# Cancer incidence among incarcerated and formerly incarcerated individuals: A statewide retrospective cohort study

**DOI:** 10.1002/cam4.6162

**Published:** 2023-05-29

**Authors:** Jenerius A. Aminawung, Pamela R. Soulos, Oluwadamilola T. Oladeru, Hsiu‐Ju Lin, Lou Gonsalves, Lisa B. Puglisi, Sirad Hassan, Ilana B. Richman, Emily A. Wang, Cary P. Gross

**Affiliations:** ^1^ Department of Internal Medicine, SEICHE Center for Health and Justice Yale School of Medicine New Haven Connecticut USA; ^2^ Department of Internal Medicine, Cancer Outcomes, Public Policy, and Effectiveness Research (COPPER) Center Yale School of Medicine New Haven Connecticut USA; ^3^ Department of Radiation Oncology University of Florida Gainesville Florida USA; ^4^ School of Social Work University of Connecticut Hartford Connecticut USA; ^5^ Research Division Connecticut Department of Mental Health and Addiction Services Hartford Connecticut USA; ^6^ Connecticut Tumor Registry, Connecticut Department of Public Health Hartford Connecticut USA

## Abstract

**Background:**

Cancer incidence among individuals with incarceration exposure has been rarely studied due to the absence of linked datasets. This study examined cancer incidence during incarceration and postincarceration compared to the general population using a statewide linked cohort.

**Methods:**

We constructed a retrospective cohort from a linkage of state tumor registry and correctional system data for Connecticut residents from 2005 to 2016, and identified cancers diagnosed during and within 12 months postincarceration. We estimated incidence rates (including for screen‐detectable cancers) and calculated the standardized incidence ratios (SIR) for the incarcerated and recently released populations, relative to the general population. We also examined cancer incidence by race and ethnicity within each group.

**Results:**

Cancer incidence was lower in incarcerated individuals (SIR = 0.64, 95% CI 0.56–0.72), but higher in recently released individuals (SIR = 1.34, 95% CI 1.23–1.47) compared with the general population, and across all race and ethnic strata. Similarly, nonscreen‐detectable cancer incidence was lower in incarcerated and higher in recently released populations compared to the general population. However, non‐Hispanic Black individuals had elevated incidence of screen‐detectable cancers compared with non‐Hispanic White individuals across all three populations (incarcerated, SIR = 1.66, 95% CI 1.03–2.53; recently released, SIR = 1.83, 95% CI 1.32–2.47; and general population, SIR = 1.18, 95% CI 1.16–1.21).

**Conclusion:**

Compared with the general population, incarcerated persons have a lower cancer incidence, whereas recently released persons have a higher cancer incidence. Irrespective of incarceration status, non‐Hispanic Black individuals have a higher incidence of screen‐detectable cancers compared with non‐Hispanic White individuals. Supplemental studies examining cancer screening and diagnoses during incarceration are needed to discern the reasons for observed disparities in incidence.

## Graphical abstract

Individuals recently released from incarceration were found to have a higher cancer incidence compared with the general population. Non‐Hispanic Black individuals had a higher incidence of screen‐detectable cancers compared with non‐Hispanic White individuals, irrespective of incarceration exposure.

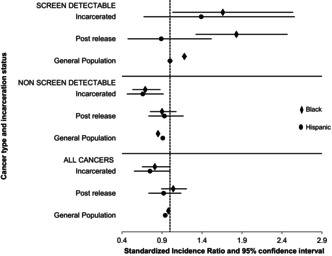

## 1 INTRODUCTION

Cancer remains a leading cause of morbidity and mortality in the United States (US) with an estimated two million Americans receiving a cancer diagnosis in 2021.^1^ Although age‐standardized cancer incidence rates in the United States have decreased over the past decades, racial disparities in cancer incidence persist. Most reports have shown Black males to have a higher cancer incidence, especially for common and screen‐detectable cancer types, compared with White males.^2^, ^3^, ^4^ Black individuals have the highest incidence of colorectal cancer among all US racial and ethnic groups.^5^ Several factors including poverty, residential segregation, and poor access to health care are associated with increased burden of cancer and poor outcomes.^6^, ^7^ These factors are driven by structural racism and thus are common among individuals who have interacted with institutions that contribute to structural racism, such as the criminal legal system.

Given that one in three Black men in the United States will be incarcerated in their lifetime,^8^ the disproportionate incarceration of Black men has important implications for racial disparities in cancer. Several studies have reported a higher cancer prevalence among incarcerated and formerly incarcerated individuals, compared with the general population.^9^, ^10^, ^11^ Also, cancer has become the number one cause of illness‐related deaths in US prisons.^12^ Factors unique to correctional systems may contribute to the higher burden of cancer in individuals with criminal justice involvement. While health care is constitutionally guaranteed during incarceration, incarcerated individuals (in Canada and other settings) often do not receive recommended cancer screening or experience significant delays in screening during incarceration.^13^, ^14^, ^15^ The immediate postrelease period is also potentially fraught with health risks, as recently released individuals frequently have no insurance, fewer economic resources, competing priorities, and poor access to care.^16^, ^17^

As such, Black and Hispanic individuals may experience a higher incidence of cancer given their disproportionate exposure to the carceral system.

Cancer incidence among individuals who have experienced incarceration has been sparsely studied due to the absence of appropriately linked datasets.^18^ Prior work that has described the association between incarceration and cancer incidence did not distinguish between cancers diagnosed while incarcerated or in the community following the index incarceration.^19^, ^20^ One prior study noted a higher incidence of cancer among individuals with a history of incarceration,^19^ while another found a similar incidence to the general population.^20^ However, these studies could not ascertain whether cancer diagnosis occurred before, during, or after release from incarceration, or assess this relationship across strata of race or ethnicity. To address these knowledge gaps, we created a novel linkage between state cancer registry and incarceration data to estimate cancer incidence among people with criminal legal system exposure, both during incarceration and in the postrelease period, compared to the general population. We also examined cancer incidence by race and ethnicity during incarceration, the immediate postrelease period and in the general population.

## 2 METHODS

### 2.1 Study design and data sources

We created a population‐based retrospective cohort through a linkage of administrative data from the Connecticut Tumor Registry (CTR) and Connecticut Department of Correction (CT DOC) from 2005 to 2016 to examine cancer incidence among incarcerated and recently released individuals in Connecticut. CT DOC has a unified criminal justice system where jails and prisons are under the authority of a single agency. Our linkage procedures and data sources have been described elsewhere.^21^ In short, we identified individuals who had experienced incarceration from the CT DOC master files between 2005 and 2016 and linked their information to the CTR using name, date of birth, sex, self‐identified race and ethnicity, and social security number. The Yale University Institutional Review Board, the Connecticut Department of Public Health Human Investigations Committee, and the CT DOC Research Advisory Council approved the study.

### 2.2 Study sample

Our study sample consisted of three populations—individuals incarcerated in a Connecticut correctional facility, individuals recently released from incarceration, and the general CT population. For individuals who experienced incarceration as identified through the CT DOC Masterfile, we used the CT DOC movement file, which delineates dates of incarceration and release, to determine if they were diagnosed during or following release from incarceration. Information on the general population in Connecticut was obtained through SEER*Stat. The sample was restricted to individuals ages 20 years and older with known sex, race, and ethnicity. Participants were categorized into the following race and ethnicity categories: Hispanic, non‐Hispanic Black, non‐Hispanic White, and other, including Asian/Pacific Islander and American Indian/Alaska Native. Due to small sample sizes in the incarcerated and recently released populations, results stratified by race and ethnicity are not presented for the “other” race category; however, when results are presented for all races and ethnicities combined, individuals in the “other” race category are included.

### 2.3 Estimation of cancer incidence rates

Among each of the three populations of interest, we estimated the incidence of invasive primary cancers diagnosed between 2005 and 2016. We calculated the incidence of all invasive cancers combined, screen‐detectable cancers—cancers with potential of early detection through specific population‐based screening recommended modalities (prostate, female breast, cervical, and colorectal cancers), and nonscreen‐detectable cancers (all other sites). We did not include lung cancer as a screen‐detectable cancer, because lung cancer screening had not yet disseminated into routine clinical practice during the study period. Information on the construction of the denominators for each population is provided in Table S1. For the incarcerated and released populations, we only had access to the month and year of diagnosis and thus assigned everyone a diagnosis date of the 15th of the month. We compared their assigned diagnosis date(s) with dates of incarceration and release to determine whether they were diagnosed while incarcerated or within 12 months of release from incarceration. A sensitivity analysis using the first, fifteenth, and thirtieth day of the month as the date of cancer diagnosis did not find any substantial differences.

### 2.4 Statistical analysis

We estimated the crude cancer incidence rates, per 100,000 person‐years, overall and by race and ethnicity.^22^ We calculated 95% confidence intervals using the exact method based on the inverse of the chi‐squared distribution.^23^ While we originally intended to calculate age‐ and sex‐standardized incidence rates using the direct method of standardization, there were many age and sex strata in the incarcerated and recently released populations that were sparsely populated, leading to unstable estimates of incidence.^24^, ^25^, ^26^ Therefore, we elected not to present directly standardized rates. For statistical comparisons across groups, we calculated the age‐ and sex‐standardized incidence ratios for the incarcerated and recently released populations, relative to the general CT population, and for Hispanic and non‐Hispanic Black individuals relative to non‐Hispanic White individuals within each incarceration group. All analyses were performed using SEER*Stat version 8.3.9.1 (SEER Research Plus Data November 2020 submission) and SAS version 9.4.

## 3 RESULTS

Our cohort included 200,983 individuals who were incarcerated, and 284,908 individuals recently released from incarceration during the period 2005–2016. Individuals who had experienced incarceration were more likely to be younger, male, and self‐identify as non‐Hispanic Black or Hispanic compared with the general population (Table 1). A cancer diagnosis was reported in 251 incarcerated individuals, 498 recently released individuals, and 244,282 individuals in the general CT population. The top three cancers were hematologic, lung, and liver and bile duct for people diagnosed while incarcerated; liver and bile duct, lung, and prostate cancer for people diagnosed postrelease; and breast, prostate, and lung cancer in the general population.

**TABLE 1:** Selected demographic characteristics of study population by incarceration status.

Characteristic	General Population *N* = 31,898,857		Incarcerated *N* = 200,983		Recently released *N* = 284,908	
	N	%	N	%	N	%
Age						
20–39	10,531,792	33.0	136,129	67.7	191,248	67.1
40–59	12,687,929	39.8	60,429	30.1	88,875	31.2
60–69	4,269,194	13.4	3664	1.8	4192	1.5
≥70	4,409,942	13.8	761	0.4	593	0.2
Sex						
Male	15,263,919	47.9	187,012	93.0	246,709	86.6
Female	16,634,938	52.1	13,971	7.0	38,199	13.4
Race and Ethnicity						
Non‐Hispanic White	23,887,908	74.9	63,833	31.8	115,038	40.4
Non‐Hispanic Black	2,978,051	9.3	83,371	41.5	97,188	34.1
Hispanic	3,688,907	11.6	52,401	26.1	70,188	24.6
Asian, Pacific Islander and American Indian, Alaska Native	1,343,991	4.2	1378	0.7	2494	0.9

For all invasive cancers, the crude incidence (per 100,000 person‐years) among the incarcerated, recently released, and general population was 124.9 (95% CI 109.9–141.3), 257.1 (95% CI 235.0–280.7), and 765.8 (95% CI 762.8–768.8), respectively. Nonscreen‐detectable cancer incidence was 101.5 (95% CI 88.0–116.4) in the incarcerated, 206.5 (95% CI 186.7–227.7) in the recently released, and 467.4 (95% CI 465.1–469.8) in the general population. Incidence of screen‐detectable cancers was 23.4 (95% CI 17.2–31.1), 50.6 (95% CI 41.1–61.6), and 284.6 (95% CI 282.8–286.5), in the incarcerated, recently released, and general population, respectively (Table 2).

**TABLE 2:** Cancer incidence by cancer type, race and ethnicity, and place of diagnosis.

	General population	Incarcerated		Recently released	
	Incidence rate^a^	Incidence rate^a^	SIR^b^	Incidence rate^a^	SIR^b^
All invasive cancers					
All races	765.8 (762.8–768.8)	124.9 (109.9–141.3)	0.64 (0.56–0.72)***	257.1 (235.0–280.7)	1.34 (1.23–1.47)***
Non‐Hispanic White	871.1 (867.4–874.9)	186.4 (154.4–223.1)	0.70 (0.58–0.83)***	318.3 (280.0–360.4)	1.33 (1.17–1.51)***
Non‐Hispanic Black	582.3 (573.7–591.1)	102.0 (81.4–126.1)	0.57 (0.46–0.71)***	245.1 (208.8–285.9)	1.35 (1.15–1.57)***
Hispanic	418.6 (412.0–425.2)	85.9 (62.6–114.9)	0.59 (0.43–0.79)***	178.1 (142.3–220.2)	1.31 (1.05–1.62)*
Nonscreen‐detectable cancers					
All races	467.4 (465.1–469.8)	101.5 (88.0–116.4)	0.75 (0.65–0.86)***	206.5 (186.7–227.7)	1.59 (1.43–1.75)***
Non‐Hispanic White	538.2 (535.2–541.1)	162.9 (133.1–197.4)	0.90 (0.73–1.09)	264.6 (229.8–303.2)	1.64 (1.43–1.88)***
Non‐Hispanic Black	312.7 (306.4–319.1)	76.8 (59.1–98.0)	0.73 (0.56–0.94)**	180.1 (149.2–215.5)	1.70 (1.41–2.04)***
Hispanic	249.1 (244–254.2)	66.8 (46.5–92.9)	0.65 (0.45–0.91)**	150.9 (118.0–190.0)	1.58 (1.24–1.99)***
Screen‐detectable cancers					
All races	284.6 (282.8–286.5)	23.4 (17.2–31.1)	0.41 (0.30–0.54)***	50.6 (41.1–61.6)	0.86 (0.70–1.05)
Non‐Hispanic White	316.9 (314.7–319.2)	23.5 (13.2–38.8)	0.28 (0.16–0.47)***	53.7 (38.7–72.6)	0.71 (0.51–0.96)*
Non‐Hispanic Black	261.4 (255.7–267.3)	25.2 (15.6–38.5)	0.36 (0.22–0.55)***	65.1 (47.1–87.6)	0.89 (0.64–1.19)
Hispanic	162.7 (158.6–166.9)	19.1 (9.2–35.1)	0.47 (0.22–0.86)**	27.2 (14.5–46.6)	0.86 (0.38–1.21)

*p ≤ 0.05; **p ≤ 0.01; ***p ≤ 0.001.

^a^ Crude cancer incidence rate per 100,000 person‐years, and 95% confidence interval.

^b^ SIR = Age‐ and sex‐standardized incidence ratio relative to general population, with 95% confidence interval.

Compared with the general population, age‐ and sex‐standardized cancer incidence for all invasive cancers was significantly lower in incarcerated individuals (standardized incidence ratio (SIR) = 0.64, 95% CI 0.56–0.72). This finding held true within all racial and ethnic strata for nonscreen‐and screen‐ detectable cancers with non‐Hispanic Black, non‐Hispanic White, and Hispanic incarcerated individuals having lower cancer incidence compared with their peers in the general population (Table 2). Conversely, among recently released individuals, cancer incidence rates were higher compared with the general population for all invasive cancers (SIR = 1.34, 95% CI 1.23–1.47), and nonscreen‐detectable cancers (SIR = 1.59, 95% CI 1.43–1.75). Screen‐detectable cancer incidence was not significantly different in the recently released group compared to the general population, except for non‐Hispanic White individuals who had a lower incidence (SIR = 0.71, 95% CI 0.51–0.96) (Table 2).

**FIGURE 1:**
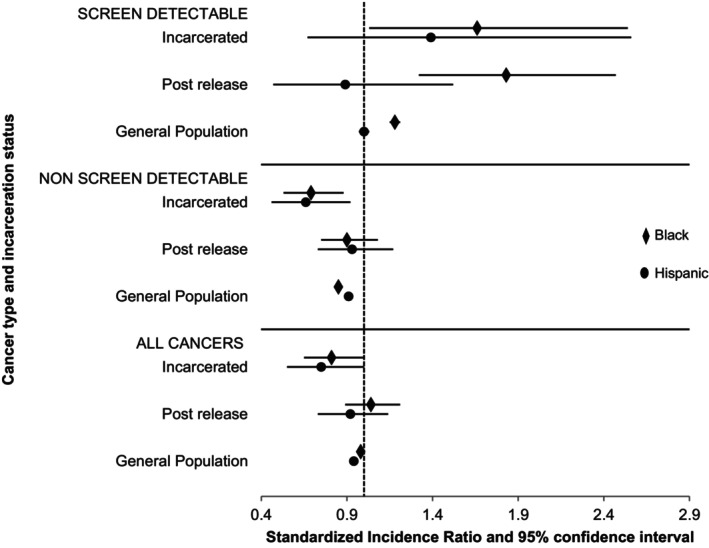
Age‐ and sex‐ standardized incidence ratios by cancer type and place of diagnosis compared to non‐Hispanic White populations.

In the general population, Hispanic (SIR = 0.98, 95% CI 0.96–0.99) and non‐Hispanic Black persons (0.94, 95% CI 0.93–0.96) had lower cancer incidence compared with non‐Hispanic White persons for all invasive cancers, with a similar trend observed for nonscreen‐detectable cancers (Figure 1). Among incarcerated adults, this same trend was observed, where Hispanic (SIR = 0.81, 95% CI 0.64–1.00) and non‐Hispanic Black (SIR = 0.75, 95% CI 0.55–1.00) persons had lower cancer incidence compared with non‐Hispanic White persons. In the recently released population, cancer incidence for all invasive and nonscreen‐detectable cancers was not significantly different between racial and ethnic groups. Within the general population, incidence of screen‐detectable cancers among non‐Hispanic Black persons was higher compared with non‐Hispanic White persons (SIR 1.18 95% CI 1.16–1.21). This pattern for screen‐detectable cancer rates was consistent among the incarcerated (SIR 1.66, 95% CI 1.03–2.53) and recently released (SIR 1.83, 95% CI 1.32–2.47) non‐Hispanic Black populations compared with non‐Hispanic White populations (Figure 1).

## 4 DISCUSSION

We observed lower cancer incidence rates among incarcerated individuals compared with the general population for all invasive cancers, while individuals recently released from incarceration had higher cancer incidence. This finding was driven by elevated rates on nonscreen‐detectable cancers in the postrelease period and persisted across all racial and ethnic strata for all invasive and nonscreen‐detectable cancers. It is important to note that cancer incidence in this context, defined by a new documented cancer diagnosis, is a function of cancer risk, access to health care, and screening utilization. Hence, possible explanations for lower incidence during incarceration include less access to routine preventive health care, less health care utilization,^27^ and delayed access to health care for symptomatic ailments within correctional systems as compared to within the community.^28^ Meanwhile, regaining insurance coverage upon release in a state that was an early adopter of Medicaid expansion, has been shown to increase rates of coverage among justice‐involved individuals.^29^ As such, might have contributed to increased health care utilization in the immediate postrelease period for unresolved physical symptoms during incarceration. Regardless of the reasons that underlie these findings, that we found a higher cancer incidence in individuals recently released from incarceration compared with the general population is disturbing given the challenges already faced by this population as they transition back into the community.

Meanwhile, cancers for which there were established screening modalities for early detection had lower incidence rates among those incarcerated and recently released from incarceration compared with the general population. These lower rates may be a reflection of low screening in both the incarcerated and postrelease populations. Factors such as lack of access to screening due to security concerns, cost of screening modalities in stretched correctional health budgets, lack of insurance coverage for incarcerated persons, and staffing constraints, may contribute to low screening rates during incarceration. Additionally, though correctional systems are constitutionally required to provide health care to individuals under their custody, institutional policies such as co‐pays may deter individuals from seeking care.^30^ Also, mistrust in the health system, competing social needs with a lesser priority on health care and delays in seeking care could contribute to the observed lower incidence of screen‐detectable cancer in the immediate postrelease period.^31^, ^32^

The incidence of screen‐detectable cancers was higher in non‐Hispanic Black persons compared with non‐Hispanic White persons in the general population. This pattern of disparity persisted in the justice‐involved population, with non‐Hispanic Black populations having a higher incidence of screen‐detectable cancer compared with non‐Hispanic White populations, despite the observed lower incidence of screen‐detectable cancers in this group. Our findings are likely driven by high incidence of prostate and colon cancer in our study population and comports with previous findings among Black adults in general.^33^ These differences have also been attributed to differences in the prevalence of factors that contribute to underlying cancer risk such as low socioeconomic status, diet and lifestyle, and poor access to colorectal cancer screening, and follow up.^34^, ^35^

### 4.1 Strengths and limitations

By using data linked from a state tumor registry to CT DOC data, we were able ascertain whether a cancer diagnosis was established during incarceration or after release, which has been missing in prior studies describing cancer incidence in those who experience incarceration.^19^, ^20^ In addition to comparing cancer incidence between individuals who have experienced incarceration and the general population, we also examined the difference by race and ethnicity within each group.

Our study does have some limitations. We only received month and year of cancer diagnosis and thus assigned all incarcerated and recently released individuals the 15th of the month as day of diagnosis. However, a sensitivity analysis using the first, fifteenth, and thirtieth day of the month as the date of cancer diagnosis, did not show any significant difference in our group allocation. We did not distinguish incarceration in jails and prisons, given that CT has a unified correctional system that includes both prisons that house individuals with longer sentences and jails that house those on pretrial or with shorter sentences.^36^ A transitory jail population may not allow enough time for symptom assessment, which could contribute to the lower incidence during incarceration and higher incidence postrelease. We used the general CT population as the comparator for cancer incidence in the incarcerated and recently released populations, which includes cancer cases diagnosed among incarcerated and released individuals, as it was not feasible to create a comparator group that consisted solely of CT residents without a history of incarceration. This approach has been used previously.^19^ However, internal analysis showed that only 0.5%–1.7% of cancer cases in CT occurred in current or formerly incarcerated individuals during the study period. Thus, keeping these individuals in the general population does not appreciably impact our results and conclusions. Cancers were categorized in our study as screen‐detectable because there were screening modalities in practice to enable early detection, and not because they were diagnosed through screening. Finally, we did not have access to patient‐level cancer risk data. Future work should explore the prevalence and impact of factors such as smoking, hepatitis C, HPV vaccine, and prior screening use, for example.

### 4.2 Conclusion

We found that individuals who are incarcerated have a lower cancer incidence compared with the general population, while those who are recently released from incarceration have a higher cancer incidence. Additionally, Non‐Hispanic Black persons have a higher incidence of screen‐detectable cancers compared with Non‐Hispanic White persons, regardless of incarceration status. These findings underscore a delay in diagnosis and the need for improved cancer screening and detection in a disproportionally minority incarcerated population in order to improve cancer inequities. Supplemental studies examining cancer screening and diagnoses during incarceration are needed to discern the reasons for observed disparities in incidence.

## AUTHOR CONTRIBUTIONS

**Jenerius Aminawung:** Conceptualization (equal); methodology (equal); project administration (equal); writing – original draft (lead); writing – review and editing (equal). **Pamela Soulos:** Conceptualization (equal); formal analysis (lead); methodology (equal); writing – review and editing (equal). **Oluwadamilola T. Oladeru:** Conceptualization (equal); methodology (equal); writing – review and editing (equal). **Hsiu‐Ju Lin:** Conceptualization (equal); data curation (lead); methodology (equal); writing – review and editing (equal). **Lou Gonsalves:** Conceptualization (equal); writing – review and editing (equal). **Lisa Puglisi:** Conceptualization (equal); methodology (equal); writing – review and editing (equal). **Sirad Hassan:** Project administration (equal); writing – review and editing (equal). **Ilana B. Richman:** Methodology (equal); writing – review and editing (equal). **Emily Wang:** Conceptualization (equal); funding acquisition (equal); methodology (equal); supervision (equal); writing – original draft (equal); writing – review and editing (equal). **Cary Gross:** Conceptualization (equal); funding acquisition (equal); methodology (equal); supervision (equal); writing – review and editing (equal).

## FUNDING INFORMATION

This work is supported by the National Institutes of Health R01 5R01CA230444–02, awarded to Dr. Cary Gross and Dr. Emily Wang. The Connecticut Tumor Registry is supported by federal funds from the National Cancer Institute, National Institutes of Health, Department of Health and Human Services, under Contract No. HHSN261201800002I.

## CONFLICT OF INTEREST STATEMENT

Dr. Oladeru reports funding unrelated to submitted work from Radiation Oncology Institute, NRG Oncology and Bristol Meyers Squibb Foundation. Dr. Gross has received research funding from NCCN Foundation (funds provided by AstraZeneca), and Genentech as well as funding from Johnson and Johnson to help devise and implement new clinical trial data sharing approaches. Dr. Richman reports salary support unrelated to the submitted work from the Centers for Medicare and Medicaid Services to develop health care quality measures. Ms. Soulos reports consulting fees unrelated to the submitted work from TARGET Pharma Solutions, Inc.

## ETHICS STATEMENT

The Yale University Institutional Review Board, the Connecticut Department of Public Health Human Investigations Committee and the CT DOC Research Advisory Council approved the study. Participant consent was waived by Yale University Institutional Review Board, and the Connecticut Department of Public Health Human Investigations Committee.

## Supporting information


Table S1

## Data Availability

The data that support the findings of this study are available from the Connecticut Department of Public Health, through a formal request and approval from the human investigations committee of the Connecticut Department of Public Health (dph.hic@ct.gov).
