# Supplementary material for: Cancer incidence among incarcerated and formerly incarcerated individuals: A statewide retrospective cohort study
Source: Cancer Med. 2023 May 29;12(14):15447–54. doi: 10.1002/cam4.6162 (PMC10417084; doi:10.1002/cam4.6162)
Supplement: Supplementary file 1 — Table S1 [file CAM4-12-15447-s001.docx]

**Appendix**

Table S1. Approach for estimating cancer incidence numerators and denominators by population.

| **Population** | **Numerator** | **Denominator** |
| --- | --- | --- |
| Incarcerated | Number of primary invasive cancers diagnosed during incarceration, 2005-2016 | Sum of individuals incarcerated on July 1 of each year, 2005-2016 |
| Recently released | Number of primary invasive cancers diagnosed within 12 months of release, 2005-2016 | Sum of individuals discharged from a correctional facility each year, 2005-2016. Denominator was discounted by 32% to account for recidivism within 12 months^†^ |
| General population | Number of primary invasive cancers diagnosed in CT, 2005-2016 | Sum of population estimates on July 1 of each year, 2005-2016 |

† Connecticut Office of Policy and Management (OPM) Criminal Justice Policy & Planning Division. Recidivism, 2017-release cohort. <https://www.jrsa.org/pubs/sac-digest/vol-32/ct-recidivsm-report-2020.pdf>.
